# Lifestyle and glycemic health 5 years postpartum in obese and non-obese high diabetes risk women

**DOI:** 10.1007/s00592-020-01553-1

**Published:** 2020-07-25

**Authors:** Emilia Huvinen, Elina Engberg, Jelena Meinilä, Tuija Tammelin, Janne Kulmala, Kati Heinonen, Paula Bergman, Beata Stach-Lempinen, Saila Koivusalo

**Affiliations:** 1 Department of Obstetrics and Gynecology, University of Helsinki and Helsinki University Hospital, Haartmaninkatu 2, PL 140, 00029 HUS Helsinki, Finland; 2 Folkhälsan Research Center, Helsinki, Finland; 3 Clinicum, Faculty of Medicine, University of Helsinki, Helsinki, Finland; 4 Department of Food and Nutrition, University of Helsinki, Helsinki, Finland; 5 LIKES Research Centre for Physical Activity and Health, Jyväskylä, Finland; 6 Department of Psychology and Logopedics, University of Helsinki, Helsinki, Finland; 7 Biostatistics Consulting, Department of Public Health, University of Helsinki and Helsinki University Hospital, Helsinki, Finland; 8 Department of Obstetrics and Gynecology, South-Karelia Central Hospital, Lappeenranta, Finland

**Keywords:** Gestational diabetes, Heterogeneity, Type 2 diabetes, Physical activity, Diet

## Abstract

**Aim:**

Women with prior gestational diabetes (GDM) are at increased diabetes risk. This study aimed to assess whether lifestyle is associated with glycemic health of high-risk women 5 years postpartum, taking into account the pre-pregnancy BMI.

**Methods:**

The RADIEL study enrolled before or in early pregnancy 720 women with pre-pregnancy BMI ≥ 30 kg/m^2^ and/or prior GDM. The follow-up visit 5 years postpartum included questionnaires and measurements of anthropometrics, blood pressure, and physical activity (PA) as well as analyses of glucose metabolism, lipids, and inflammatory markers. We measured body composition (Inbody) and calculated a Healthy Food Intake Index (HFII) from Food Frequency Questionnaires (FFQ). ArmBand measured PA, sedentary time, and sleep. To take into account the diverse risk groups of GDM, we divided the women based on pre-pregnancy BMI over/under 30 kg/m^2^.

**Results:**

Altogether 348 women attended the follow-up. The obese and non-obese women showed similar prevalence of glycemic abnormalities, 13% and 19% (*p* = 0.139). PA levels were higher among the non-obese women (*p* < 0.05), except for step count, and their HFII was higher compared to the obese women (*p* = 0.033). After adjusting for age, education, and GDM history, PA and HFII were associated with glycemic health only among obese women. When both lifestyle factors were in the same model, only PA remained significant. PA associated with other markers of metabolic health also among the non-obese women, excluding HbA1c.

**Conclusion:**

Lifestyle 5 years postpartum was associated with better glycemic health only among the obese high-risk women. PA, however, is essential for the metabolic health of all high-risk women.

**Clinical trial registration:**

ClinicalTrials.gov, http://www.clinicaltrials.com, NCT01698385.

## Introduction

The escalating epidemic of obesity and diabetes [1] requires efficient methods for prevention. Fortunately, according to large international studies, type 2 diabetes (T2D) is preventable among high-risk groups with lifestyle intervention [2, 3]. Women with a history of gestational diabetes (GDM) are at a sevenfold risk of developing future diabetes [4, 5] and therefore many intervention studies have recently focused also on women with prior GDM [6]. For example, the RADIEL (The Finnish Gestational Diabetes Prevention) lifestyle intervention starting during pregnancy and continuing during the 1st postpartum year successfully reduced the incidence of glycemic abnormalities 1 year after delivery [7].

T2D and GDM, however, are heterogeneous diseases. Scandinavian researchers recently categorized T2D into 5 subclasses [8], each with varying characteristics, complications, and progression. The heterogeneity of GDM has received less interest, but in addition to T2D, also women with GDM differ by their body composition, insulin secretion and resistance, as well as diabetes-related autoimmunity [9–12]. In the RADIEL study, the incidence of glycemic abnormalities was high 5 years postpartum also among the non-obese women with prior GDM, and they presented often with normal-weight obesity (NWO), i.e., normal body mass index (BMI) but high body fat percentage [13].

There are suggestions that physical inactivity, smoking, alcohol consumption, and poorer dietary habits of NWO individuals could explain their impaired metabolic health [14]. This could offer one possible explanation for the high prevalence of glycemic abnormalities postpartum also among the non-obese GDM women. On the other hand, non-obese women with GDM have typically a deficiency in insulin production [15] and we hypothesize that lifestyle changes aiming at reducing insulin resistance by increasing physical activity (PA) and improving diet quality might not have similar effect among non-obese and obese women. The aim of this study was to assess the lifestyle of women at high risk for diabetes 5 years postpartum and to evaluate the associations between lifestyle and glycemic health in GDM subgroups, i.e., obese and non-obese women based on their pre-pregnancy BMI over/under 30 kg/m^2^.

## Materials and methods

### Study design

This is a cross-sectional study focusing on the participants of the RADIEL 5-year follow-up study. Originally, the RADIEL lifestyle intervention study was a randomized controlled trial (RCT), recruiting women at high risk for GDM before or in early pregnancy. Altogether 724 women entered the study between years 2008–2014. During years 2013–2017, 5 years after delivery, all participants with a live birth and their children were invited to a follow-up study. In the original study, some participants visited the study nurse for data collection every 3 months before pregnancy and all participants once in each trimester of pregnancy and 6 weeks, 6 and 12 months postpartum. The study visits took place in Helsinki, in the Helsinki University Hospital (HUH), and in Lappeenranta, in South Karelian Central Hospital (SKCH). Previous publications provide the details of these studies [13, 16].

The study participants were at least 18 years of age and had a BMI 30 kg/m^2^ or over, and/or prior GDM. Overt diabetes, medications altering glucose metabolism, multiple pregnancy, severe psychiatric problems, physical disabilities, and communication problems based on inadequate language skills led to exclusion. All participants gave a written informed consent and the ethics committees of the HUH and SKCH both approved the study protocol.

### Outcomes

The main outcome of this study is the presence of glycemic abnormalities 5 years after delivery. All participants, except for those with physician-diagnosed T2D or prior bariatric surgery, underwent a 75 g 2-h oral glucose tolerance test (OGTT). T2D diagnosis was based on fasting plasma glucose ≥ 7.0 mmol/l or 2-h glucose ≥ 11.1 mmol/l. The definition for impaired glucose tolerance (IGT) was 2-h glucose 7.8–11.0 mmol/l and diagnosis of impaired fasting glucose (IFG) required fasting plasma glucose in the range of 6.1–6.9 mmol/l. Completing the criteria of either IFG, IGT, or T2D resulted in a composite outcome of glycemic abnormality.

Each study visit included measurements of body weight, height, and waist and hip circumference. The calculation for postpartum weight change derived from the difference between weight at the study visits 6 weeks and 5 years postpartum. Blood pressure was measured with a sphygmomanometer in a sitting position from the right arm. The laboratory tests in conjunction with the study visit, after a 10–12 h fast, included analysis of glucose metabolism (2 h 75 g OGTT, GHbA1c, insulin), lipids (cholesterol, low-density lipoprotein cholesterol LDL, high-density lipoprotein cholesterol HDL, and triglycerides), inflammatory markers (highly sensitive C-reactive protein hs-CRP), and alanine amino transferase (ALAT). A multi-frequency bio-impedance measurement method (InBody3.0, Biospace Co., Ltd, Seoul, Korea) provided the information on body composition [17]. AGE Reader measured the advanced-glycation end-products (AGEs), which are potential early markers of diabetes risk.

Lifestyle in this study refers to PA and diet. SenseWear ArmBand Pro 3 recorded PA, sedentary time, and sleep of the participants, who wore the monitor in their upper non-dominant arm. The monitor required removal while showering, sauna, and water-sports. To be included in the analysis, we required recording of at least 85% of a day, during a minimum of 4 days, and including at least one weekend day. We extracted measures for light PA (LPA) (min/day), moderate-to-vigorous PA (MVPA) (min/day), vigorous PA (VPA) (min/day), sleep (h/day), steps (counts/day), and sedentary time (min/day, excluding sleep).

Food frequency questionnaires (FFQ) provided the information on current diet. In the analysis, we used the daily intake of specified food groups as well as a healthy food intake index (HFII) describing the diet as an entity; participants received points reflecting their intake of high-energy/low-nutrient snacks (0–2 points), sugar-sweetened beverages (0–1 points), fast food (0–1 points), high-fiber grains (0–2 points), bread fat spread (0–2 points), low-fat cheese (0–1 points), low-fat milk (0–2 points), fish (0–2 points), red and processed meat (0–2 points), vegetables (0–2 points), and fruits and berries (0–1 points). The maximum score available was 18, with a higher score indicating a healthier diet. In case of a missing answer, the HFII score was not calculated. We used HFII both as continuous and as categorical, divided into three categories. The first cut-off was determined by subtracting 1SD from the mean and the second by adding 1SD to the mean.

Background questionnaires covered additional aspects of lifestyle, such as smoking, with regular and occasional smoking combined. Questionnaires also recorded self-reported duration of breastfeeding, years of education, family income as well as chronic illnesses. The Center for Epidemiological Studies Depression Scale (CES-D) [18] provided the tool for assessing depressive symptoms. In the analysis we used both the logarithmic sum as a continuous variable and the generally acknowledged cut-off of 16 points to indicate depression [19].

For assessing the associations between lifestyle and glycemic health, we divided the women based on their pre-pregnancy BMI over or under 30 kg/m^2^. The justification for using pre-pregnancy instead of current BMI was the high metabolic risk of the normal-weight women with GDM [20]. The postpartum weight change in either direction could have mixed these diverse risk groups which potentially have different pathophysiological backgrounds. When referring to subgroups based on BMI (non-obese and obese) in this article, we always refer to the pre-pregnancy BMI.

### Statistics

We examined normal distribution of the variables with the Shapiro–Wilk test. Descriptive characteristics are presented as means with standard deviations (SD), medians with interquartile range (IQR) or as frequencies with percentages. Between-groups comparisons were performed with the Chi square test, the Mann–Whitney *U* test, ANOVA, or the independent sample *T* test, when appropriate. For examining the associations between glycemic abnormalities and lifestyle (MVPA and HFII) we used the binary logistic regression model. We assessed the correlations between PA and metabolic characteristics with Spearman’s rank-order correlation. We performed the statistical analyses with the SPSS 24.0 software program (SPSS Inc., Chicago, IL, USA) and considered a *p* value < 0.05 as statistically significant.

## Results

Data on glucose metabolism were available for 348 women, HFII for 298 women, and device-measured PA for 206 women. In total 51 women (13.6%) had a glycemic abnormality; 24 women (6.4%) had IFG, 24 women (6.4%) IGT, and 13 women (3.5%) T2D.

At the follow-up visit, previously obese and non-obese women differed significantly in most measured parameters (Table 1). In addition to better metabolic health, non-obese women also showed lower depression scores. There were no differences in age, smoking, family history of diabetes, or GHbA1c between the BMI-groups. Among the obese women, 7 (3.1%) were diagnosed with T2D, 16 (7.2%) had IGT, and 13 women (5.8%) IFG. The corresponding numbers for non-obese women were 6 (4.8%), 8 (6.4%) and 11 (8.8%), respectively. There was no statistically significant difference in the prevalence of IFG, IGT, or T2D between the groups. The indices for insulin resistance (HOMA-IR) and insulin secretion (HOMA-β) were significantly different: previously obese women showed higher HOMA-IR and HOMA-β levels.

**Table 1:** Characteristics 5 years postpartum, in subgroups of obese and non-obese participants based on the pre-pregnancy BMI > 30 kg/m^2^

Variable	All 348	Obese 223	Non-obese 125	*p* value^a^
BMI (kg/m^2^)	31.7 (6.7)	35.2 (5.3)	25.5 (3.6)	< 0.001
Age (years)	38.6 (4.6)	38.4 (4.8)	39.0 (4.2)	0.249
Family history of diabetes (*n*, %)	108 (32)	63 (29)	45 (36)	0.201
Body fat percentage	39.7 (32.9, 46.1)	43.9 (39.1, 48.4)	32.1 (26.1, 36.1)	< 0.001
GDM history (*n*, %)^b^	236 (68)	111 (50)	125 (100)	< 0.001
AGE value	1.80 (1.60, 1.90)	1.80 (1.60, 1.95)	1.80 (1.55, 1.90)	0.758
Smoking (*n*, %)	48 (14)	32 (15)	16 (13)	0.749
Depression (log sum CES-D) (*n* = 179 + 94)	2.3 (1.8, 2.8)	2.4 (1.8, 2.9)	2.1 (1.4, 2.6)	0.033
Depression (CES-D > 16) (*n*, %) (*n* = 179 + 94)	75 (27.5)	55 (30.7)	20 (21.3)	0.097
Waist circumference (cm)	105 (16)	113 (14)	91 (11)	< 0.001
Hip circumference (cm)	112 (13)	118 (11)	101 (8)	< 0.001
RR systolic (mmHg)	125 (14)	129 (15)	118 (11)	< 0.001
RR diastolic (mmHg)	79 (10)	81 (11)	75 (8)	< 0.001
Total cholesterol (mmol/l)	4.7 (0.8)	4.6 (0.8)	4.8 (0.7)	0.085
HDL cholesterol (mmol/l)	1.5 (0.4)	1.4 (0.3)	1.6 (0.3)	< 0.001
LDL cholesterol (mmol/l)	3.0 (0.8)	3.0 (0.8)	3.0 (0.7)	0.812
Triglycerides (mmol/l)	0.9 (0.7, 1.2)	0.9 (0.7, 1.4)	0.8 (0.6, 1.0)	< 0.001
GHbA1c (%)	5.4 (0.5)	5.4 (0.6)	5.4 (0.5)	0.982
Fasting plasma insulin (mU/l)	9.3 (6.3, 14.4)	11.3 (7.5, 15.9)	6.7 (5.0, 9.6)	< 0.001
HOMA-IR	2.2 (1.4, 3.3)	2.7 (1.6, 3.6)	1.6 (1.1, 2.3)	< 0.001
HOMA-β (%)	116.2 (76.4, 178.8)	140.0 (96.5, 200.0)	76.7 (61.6, 116.7)	< 0.001
hs-CRP (mg/l)	1.4 (0.6, 3.3)	2.0 (1.0, 4.4)	0.6 (0.4, 1.5)	< 0.001
Postpartum weight change (%)	2.2 (− 5.1, 8.1)	5.1 (− 2.7, 10.2)	− 0.1 (− 6.4, 4.8)	< 0.001

Data are presented as means (SD), medians (IQR), or frequencies (%)

^a^Comparing obese and non-obese women. Results from Independent samples *t* test, Mann–Whitney *U* test, or Chi Square accordingly

^b^GDM history refers to GDM either before or in the RADIEL pregnancy

Table 2 presents the lifestyle measurements 5 years after delivery. The non-obese women slept more, had less sedentary time, and higher PA levels, except for the step count, which did not differ between the groups. They also reported to consume less often snacks (*p* = 0.043) and more often high-fiber grains (*p* = 0.011), and also their HFII was statistically significantly higher indicating a healthier diet compared to the obese women (*p* = 0.033).

**Table 2:** Lifestyle 5 years postpartum among previously non-obese and obese women

Lifestyle	Non-obese women	Obese women	*p* value
Physical activity (*n*)	80	126	
Sleep (h/day)	7.1 (6.5, 7.5)	6.7 (6.1, 7.3)	0.006
Sedentary time (min/day)	555 (500, 631)	658 (575, 750)	< 0.001
LPA (min/day)	269 (239, 330)	249 (181, 320)	0.024
MVPA (min/day)	89 (65, 124)	51 (34, 75)	< 0.001
VPA (min/day)	1.5 (0.1, 6.6)	0.1 (0, 1.3)	< 0.001
Steps (counts/day)	9316 (6623, 10,910)	8202 (6480, 10,287)	0.113
Diet (*n*)	108	190	
HFII (points)	10 (8, 12)	9 (8, 11)	0.033
Red and processed meats (times/day)	1.42 (0.9, 2.3)	1.6 (1.1, 3.0)	0.055
Whole-grain cereals (times/day)	2.8 (1.5, 4.0)	2.2 (1.3, 3.5)	0.044
Vegetables and fruits (times/day)	7.1 (4.5, 9.7)	6.3 (4.4, 9.4)	0.722
Snacks (times/week)	0.9 (0.6, 1.5)	1.1 (0.7, 1.6)	0.043
Sugar-sweetened beverages (times/week)	0.1 (0, 0.1)	0.1 (0, 0.2)	0.961
Fish (times/week)	0.5 (0.4, 0.7)	0.5 (0.4, 0.7)	0.500
Fast food (times/week)	0.2 (0.1, 0.2)	0.2 (0.1, 0.2)	0.689

Physical activity, sedentary time, and sleep were measured with SenseWear Armband and diet index and intakes of specific food groups were calculated from FFQs

Data are presented as medians (IQR)

*FFQ* food frequency questionnaire, *LPA* light physical activity, *MVPA* moderate-to-vigorous physical activity, *VPA* vigorous physical activity, *HFII* healthy food intake index, maximum score 18

In the total study population, there was a detectable association between glycemic abnormalities and age [crude OR 1.120 (1.044, 1.201) *p* = 0.002], years of education [crude OR 0.667 (0.449, 0.990) *p* = 0.044], body fat percentage [crude OR 1.044 (1.066, 1.085) *p* = 0.025], postpartum weight change [crude OR 1.055 (1.020, 1.090) *p* = 0.002], GDM history [crude OR 5.181 (1.998, 13.435) *p* = 0.001] and current BMI [crude OR 1.058 (1.012, 1.107) *p* = 0.012]. There was no association between glycemic abnormalities and RADIEL lifestyle intervention, depression, breastfeeding, diet, physical activity, sleep, or family income. Based on these results, we adjusted the further logistic regression models with GDM history, age, and education years. Body fat percentage and postpartum weight change were not included, as they can be considered as mediators of the lifestyle effects.

**Fig. 1:**
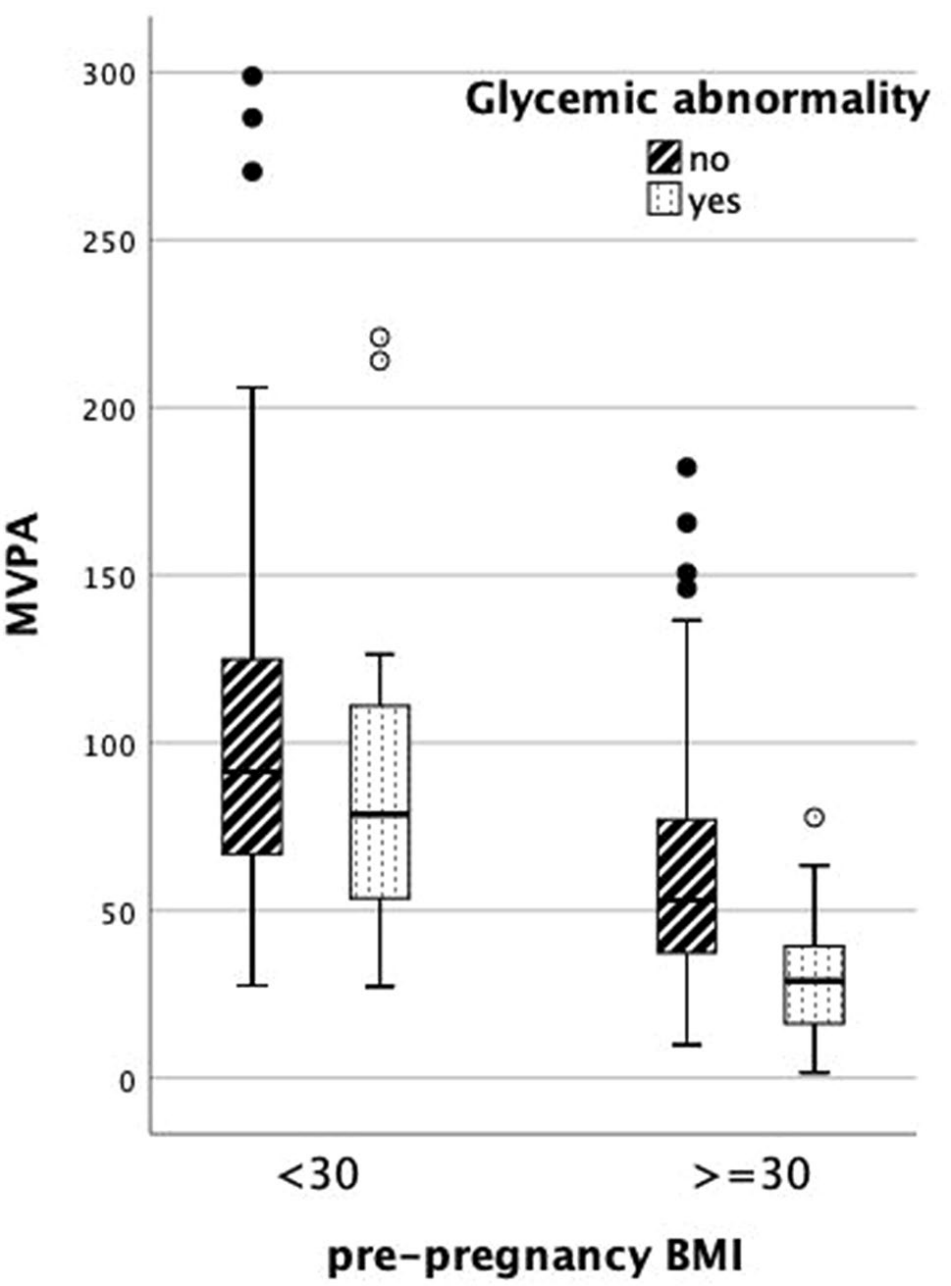
Moderate-to-vigorous physical activity (MVPA min/day) in groups of previously non-obese and obese women, according to presence of glycemic abnormalities. Interaction *p* = 0.009. For the clarity of the figure, two extremely high outliers were excluded from the group of non-obese women without glycemic abnormality (383 min/day and 360 min/day)

There was an interaction between pre-pregnancy BMI and MVPA (*p* = 0.009) on glycemic abnormalities (Fig. 1), but not between pre-pregnancy BMI and diet. Table 3 presents the multivariable regression models among obese and non-obese women. Lifestyle was associated with glycemic health only among women with pre-pregnancy BMI 30 kg/m^2^ or higher; after adjusting with age, education, and GDM history, both MVPA and HFII were associated with glycemic health. When adjusted additionally with current BMI and analyzing both lifestyle factors in the same model, only PA remained significant. When performing the regression analysis with HFII as a categorical variable the results were similar (data not shown).

**Table 3:** Logistic regression models demonstrating associations of physical activity and diet quality with glycemic abnormalities 5 years postpartum in obese and non-obese women

	(A) Logistic regression models adjusted with age, education, and GDM history											
	Model 1				Model 2				Model 3			
	BMI < 30 *n* = 80		BMI ≥ 30 *n* = 126		BMI < 30 *n* = 106		BMI ≥ 30 *n* = 190		BMI < 30 *n* = 69		BMI ≥ 30 *n* = 107	
	OR	*p*	OR	*p*	OR	*p*	OR	*p*	OR	*p*	OR	*p*
MVPA	0.99 (0.98, 1.0)	0.18	0.96 (0.93, 0.99)	0.01	–	–	–	–	0.99 (0.98, 1.0)	0.15	0.95 (0.91, 0.99)	0.02
HFII	–	–	–	–	0.98 (0.82, 1.17)	0.82	0.80 (0.65, 0.99)	0.04	1.07 (0.83, 1.38)	0.60	0.73 (0.53, 1.01)	0.05

	(B) Logistic regression models adjusted with age, education, GDM history, and current BMI											
	Model 4				Model 5				Model 6			
	BMI < 30 *n* = 80		BMI ≥ 30 *n* = 126		BMI < 30 *n* = 106		BMI ≥ 30 *n* = 190		BMI < 30 *n* = 69		BMI ≥ 30 *n* = 107	
	OR	*p*	OR	*p*	OR	*p*	OR	*p*	OR	*p*	OR	*p*
MVPA	1.00 (0.98, 1.01)	0.64	0.97 (0.94, 0.999)	0.05	–	–	–	–	0.996 (0.98, 1.01)	0.59	0.95 (0.91, 0.998)	0.04
HFII	–	–	–	–	1.09 (0.87, 1.37)	0.45	0.80 (0.64, 0.997)	0.05	1.16 (0.86, 1.57)	0.34	0.73 (0.53, 1.01)	0.06

*HFII* healthy food intake index, *MVPA* moderate-to-vigorous physical activity

To further assess the importance of PA in phenotypically diverse groups, we evaluated the correlations between measures of PA, sedentary time, and sleep and markers of metabolic health. Table 4 presents these correlations, adjusted with age, education years, and GDM history, separately among obese and non-obese women. Among obese women, VPA, MVPA, LPA, and sedentary time correlated with all markers of metabolic health. Step count correlated with body fat percentage, HDL, insulin, and blood pressure. Among non-obese women VPA and MVPA correlated with all other markers of metabolic health, except for GHbA1c.

**Table 4:** Correlations between markers of metabolic health and measures of physical activity, sleep, and sedentary time among subgroups of obese and non-obese women

PA	Body fat %	Waist	HbA1c	hs-CRP	HDL	LDL	Triglycerides	Insulin	Systolic RR	Diastolic RR
	*r*^s^	*r*^s^	*r*^s^	*r*^s^	*r*^s^	*r*^s^	*r*^s^	*r*^s^	*r*^s^	*r*^s^
Obese										
Sleep	− 0.27	− 0.109	− 0.092	− 0.062	0.115	− 0.048	− 0.095	− 0.047	0.001	0.036
Steps	− 0.200*	− 0.055	− 0.140	− 0.122	0.254*	− 0.136	− 0.123	− 0.187*	− 0.233*	− 0.239*
Sedentary	0.515**	0.427**	0.269*	0.440**	− 0.416**	0.272*	− 0.316**	0.359**	0.339**	0.421**
LPA	− 0.437**	− 0.357**	− 0.198*	− 0.399**	0.373**	− 0.196*	− 0.194*	− 0.302*	− 0.384**	− 0.473**
MVPA	− 0.437**	− 0.332**	− 0.204*	− 0.343**	0.240**	− 0.209*	− 0.287*	− 0.382**	− 0.269*	− 0.299*
VPA	− 0.496**	− 0.461**	− 0.261*	− 0.486**	0.399**	− 0.100	− 0.413**	− 0.545**	− 0.200*	− 0.276*
Non-obese										
Sleep	0.168	0.022	− 0.175	0.025	0.011	0.100	0.144	0.127	− 0.109	− 0.051
Steps	− 0.324*	− 0.227*	0.162	− 0.184	0.135	− 0.243*	− 0.232*	− 0.279*	0.054	− 0.042
Sedentary	0.300*	0.320*	− 0.046	0.223	− 0.273*	0.206	0.111	0.106	0.092	0.102
LPA	0.161	0.162	0.056	− 0.007	0.046	− 0.033	0.012	− 0.010	0.198	0.075
MVPA	− 0.673**	− 0.637**	− 0.027	− 0.445**	0.322*	− 0.255*	− 0.289*	− 0.397**	− 0.320*	− 0.195
VPA	− 0.577**	− 0.473**	0.123	− 0.341*	0.224*	− 0.235*	− 0.415**	− 0.561**	− 0.217*	− 0.238*

Spearman’s rank-order correlations adjusted for age, education, and history of gestational diabetes

*p < 0.05

**p < 0.005

*LPA* light physical activity, *MVPA* moderate-to-vigorous physical activity, *VPA* vigorous physical activity

## Discussion

The association between lifestyle and glycemic health varies between obese and non-obese high-risk women. In our study, the non-obese women were physically more active and had a healthier diet compared to obese women. Despite their healthier lifestyle, however, they presented with similar prevalence of glycemic abnormalities 5 years postpartum. When adjusted for age, education, and GDM history, PA and diet were associated with better glycemic health only among the obese women. This remained significant even when adjusted with current BMI. However, higher levels of MVPA and VPA were associated with numerous other components of metabolic health, such as lower body fat percentage, blood pressure, and healthier lipid profile, also among the non-obese high-risk women.

Although previous studies have suggested that NWO individuals might have poorer dietary habits, this was not true in our population. The non-obese women had overall a higher HFII indicating a healthier diet compared to obese participants. They also consumed less snacks and more whole grain products, which have been suspected to improve the function of beta cells [21], and there was a trend toward lower consumption of red meats, generally associated with lower diabetes risk [22]. The HFII of non-obese women, however, was not associated with glycemic health 5 years postpartum.

The non-obese participants were also physically more active. PA is generally associated with lower morbidity and mortality [23] and device-measured PA has shown even a stronger association with health benefits [24]. The influence of PA may be at least partly mediated through reductions in total and abdominal fat leading to improvements in insulin sensitivity and blood pressure, and possibly improvements in endothelial vascular function [25]. Our results are in line with previous studies as PA was associated with blood pressure, body fat percentage, lipids, waist circumference, insulin, and hs-CRP among all participants.

In our study there was, however, a significant interaction between PA and pre-pregnancy BMI when assessing glycemic abnormalities. Also a few other studies have assessed the influence of lifestyle in phenotypically different subgroups. An Indian study demonstrated that weight loss is beneficial also among non-obese individuals with family history of diabetes [26]. In preventing the metabolic syndrome (MetS), PA seems similarly effective among non-obese individuals [27], which has been demonstrated also elsewhere [28].

Nevertheless, there are studies reporting similar interactions in general population as in our study. In two large cohort studies, PA was associated with less visceral adipose tissue but this association attenuated after adjustment for BMI (32) and also the association between PA and BMI appeared weaker among non-obese individuals [29]. A study on 1109 adults, using accelerometer-measured PA, also demonstrated an interaction between BMI and association of MVPA and HbA1c [30].

On the other hand, the correlations between PA and metabolic parameters were similar among the obese and non-obese. The strongest correlation was with body fat percentage, waist circumference, and blood pressure. This is reassuring as lifestyle may influence the important cardiovascular risk factors also among the non-obese, which has been reported also previously [31]. In our study, among non-obese women, however, associations existed only with more strenuous PA whereas even lower intensity PA was associated with better metabolic outcome among the obese. These results additionally supported our findings concerning glycemic abnormalities, as HbA1c and PA were not correlated among non-obese women.

A few reasons have been suggested to explain the heterogeneous response to lifestyle. Gene-lifestyle interactions may influence the susceptibility to weight gain and effectiveness of lifestyle interventions [32, 33]. These interactions have been demonstrated in large studies, e.g., polymorphisms in the PPAR-gamma2 gene and Glu9 [33], as well as in the MTNR1B, which also decreased the effect of the RADIEL intervention in preventing GDM [34]. Polymorphisms in the PPAR-gamma2 gene, e.g., modify the effects of lifestyle on weight loss, lipids, and insulin sensitivity [33]. In the Finnish Diabetes Prevention Study certain polymorphisms in GHRL and LEPR genes modified the effect of PA on weight, waist circumference, lipids, and blood pressure, but had no effect on the progression from IGT to T2D [35]. Additionally, polymorphisms in the TNF-α gene may interfere with the anti-inflammatory effect of PA [36].

In addition to genetic polymorphisms, also other background factors might interfere with the effects of lifestyle. It is estimated that 5–6% of GDM women have some form of monogenetic diabetes (MODY) [37, 38], which will not respond to lifestyle changes similarly. Lifestyle changes might also have a stronger effect among people with insulin resistance [39]. Hypothetically, the adiposity-related insulin resistance might be more reversible than the defects in insulin secretion. In our study, the previously non-obese women showed significantly lower insulin secretion indices, generally associated with higher T2D risk [40]. Additionally, gut microbiota is known to influence the risk of developing diabetes in an interplay with diet [41].

Strengths of this study are the inclusion of phenotypically different groups of high-risk women. We also have detailed device-measured data on PA as well as detailed dietary information from FFQs and diet diaries. Additionally, having measured multiple metabolic markers, including body composition, enables us to analyze not only glycemic disorders but also other aspects of metabolic health such as body fat percentage. Limitations of our study include the number of participants, especially when divided into subgroups, and lack of a control group to enable comparison with normal unselected population. Due to our study design, all non-obese participants had a history of GDM indicating a higher risk of glycemic abnormalities also postpartum. This might potentially influence our findings. Also, all participants were of Caucasian origin and this limits the generalizability of our findings.

Although intervention studies in big populations have reached well-documented positive results [6, 42], it is important to assess components of lifestyle individually and in phenotypically distinct groups to further develop our recommendations for high-risk individuals. Our study demonstrated that healthy lifestyle is essential for high-risk women, but among non-obese women it was not associated with better glycemic health. PA, however, has a positive association with many other markers of metabolic health also among the non-obese, and is therefore most probably beneficial for their cardiovascular health.

We showed that higher levels of PA are associated with better metabolic health. However, other factors than lifestyle may play a role for the glycemic health of non-obese women. It is important to recognize this high-risk group of non-obese women with prior GDM, as even their physically active lifestyle and healthier diet is not a marker of lower diabetes risk. Equally important is to study the factors behind their increased risk and to find new innovative ways to prevent T2D. There is a need for precision medicine approach in diabetes research and treatment [8, 43] and studies focusing on genetic polymorphisms, microbiome, and metabolomics might provide new insights in the future. On the other hand, even more emphasis should be put on motivational lifestyle interventions among the obese women as T2D could be prevented. Perhaps individualized counseling, social support, self-efficacy, and listening to women’s wishes [44] require further attention to improve the results of lifestyle interventions [45]. As T2D is an expanding epidemic, we cannot afford to neglect this group of high-risk women.

## Data Availability

The datasets generated during and/or analyzed during the current study are available from the corresponding author on reasonable request.
